# Conceptualizing moral health for military moral injury care: theoretical foundations of the moral engagement group

**DOI:** 10.3389/fpsyt.2026.1761744

**Published:** 2026-06-04

**Authors:** Chris J. Antal

**Affiliations:** Chaplain Service, Corporal Michael J. Crescenz Veterans Affairs (VA) Medical Center, Philadelphia, PA, United States

**Keywords:** community, moral challenges, moral disengagement, moral exploitation, moral functioning, moral health, moral injury, moral overload

## Introduction

This paper presents the theoretical foundations of an existing intervention for moral injury (MI), the Moral Engagement Group (MEG) in Philadelphia, Pennsylvania (1). Previously, Antal et al. introduced the Moral Injury Group (MIG) as a “chaplain-psychologist collaboration” (2) and “community intervention” (3). MEG utilized the group protocol in the context of current controversies in military MI care (4). Cenker et al. reported positive group outcomes (5). The MIG is now called the MEG to better describe the group’s purpose, and despite the name change, the protocol itself has not changed. The aim here is to articulate a coherent moral theory conceptualizing: (1) moral health and moral functioning; (2) MI as moral challenge (demands growth) not as a moral problem (demands fixing); (3) moral disengagement, moral exploitation, and moral overload as impairments not in individual functioning but rather as moral community functioning. Without conceptual clarity, figuring out thresholds for impairment remain elusive, and experts preoccupied on correct application of therapeutic technique to fix moral problems and improve individual functioning risk overlooking the vital role moral community plays in addressing moral challenges, and could sabotage moral health and moral functioning in the process.

## Moral health and moral functioning

Drescher and Farnsworth stress MI investigators need to first “understand morality, including the contexts in which it operates and the social functions it serves” and offered “a social–functional perspective on morality and moral injury” (6), a theory also endorsed by Litz (7). Previously Antal et al. emphasized the importance of investigating contexts, including the context Herman called the “social ecology of violence” (8), from a public health perspective, to grasp the phenomenon called MI. Our focus here is a dimension of public health -- moral health-- which needs definition. For Settimo moral health “describes the impact that our moral behavior has on our own health” and consists of “the state of well-being that encompasses an individual’s moral values, principles, and behavior, influenced by their neurobiological nature, emotions, and experiences, and impacting their overall physical and mental health” (9). This definition is a good start but overlooks the vital role of moral community. Talbert and Wolfendale offer “moral health should refer to the capacities, traits, and social conditions that make it possible for persons to act as moral agents, and to be regarded by others as such.” They call this “the ability to participate in the moral community”.

The moral community is not a geographically bounded community. Rather, it is the community of persons who are appropriate objects of the reactive attitudes (emotions such as resentment and gratitude that are reactions to the quality of will expressed in others’ behavior) and therefore regarded by themselves and others as morally responsible agents (10).

A healthy moral community, then, is a community where one’s moral agency is given appropriate moral regard by others and people experience, what Wolfendale calls moral security.

Broadly speaking, a person possesses *subjective* moral security when she believes that her basic interests and welfare will be accorded moral recognition by others in her community and by social, political, and legal institutions in her society. She possesses *objective* moral security if, as a matter of fact, her interests and welfare are regarded by her society as morally important—for example, when violent crimes against her are taken to warrant the same punishment and condemnation as equivalent crimes against others (11).

Thus, moral health is not only “the state of well-being that encompasses an individual’s moral values, principles, and behavior” described by Settimo. Moral health requires both the existence of, and the ability to participate in, the moral community where people are appropriate objects of the reactive attitudes, or moral emotions (12), where moral security is assured, and where people can recognize, appropriately engage with, and work through, moral challenges together. Settimo acknowledges the importance of community in moral repair yet fails to define moral health in a way that goes beyond individual well-being to include community.

Achieving moral health as described above requires moral agents exhibit unimpaired moral functioning, an achievement that takes the intrapersonal and interpersonal work of moral engagement. The intrapersonal work includes cultivating moral literacy (13) and nurturing moral courage (14). The work of moral engagement includes exploring moral values (i.e., compassion, fairness, honesty, respect, responsibility) and moral emotions (i.e., gratitude, pride, disgust, resentment, guilt, indignation). A morally literate and morally courageous person has capacity to feel the range of pleasant and unpleasant moral emotions and rise to moral challenges. So it may be said that person exhibits unimpaired moral functioning and contributes to the moral security of others.

The interpersonal work of moral engagement requires a moral agent to draw from the experience of moral emotions the motivation to act, providing others with appropriate moral regard. Practically speaking, this could mean saying “thank you” to express gratitude for praiseworthy behavior; and “I’m sorry,” to express remorse for blameworthy behavior. Giving moral regard also includes accepting what Mellema calls moral accountability: acknowledging, where appropriate, personal complicity in, and accountability for, harmful actions they contribute to even though others may be the principal agents (15). Kutz calls this the Complicity Principle: “a person is accountable for what others do when he or she intentionally participates in the wrong they do or the harm they cause” (16).

## MI as moral challenge

MI initially emerged to describe a particular kind of veteran experience, and the term has attracted much attention, from authors in multiple disciplines, with new applications, beyond the veteran population, to include first responders, healthcare workers, refugees, and others. Even so, the construct remains problematic and any discussion of it ought to begin with what Litz calls “epistemic humility” (17). Kinghorn named the risk of approaching MI as a clinical problem:

moral injury beckons beyond the constraints of contemporary psychology toward something like moral theology. This something, embodied in specific communal practices, can rescue moral injury from the medical model and the means-end logic of *techne* and can allow for truthful contextualized narration of morally fragmenting combat experiences.

These veterans need the support of a community that can listen, reflect, bear and grieve with them. … And they need a community that is willing to own and acknowledge its own violence, as embodied in the lives and actions of its soldiers (18).

Kinghorn’s call to “rescue moral injury from the medical model” is echoed by Frame, who observes, “the field of moral injury has plainly been ‘colonized’ by psychology” (19). These authors seem to be arguing against an individualized medical model of disability and for something more grounded in a public health framework-- a social model of disability. Here are the key differences:

The medical model of disability is presented as viewing disability as a problem of the person, directly caused by disease, trauma, or other health condition which therefore requires sustained medical care provided in the form of individual treatment by professionals.

The social model of disability sees the issue of “disability” as a socially created problem and a matter of the full integration of individuals into society. In this model, disability is not an attribute of an individual, but rather a complex collection of conditions, many of which are created by the social environment. Hence, the management of the problem requires social action and is the collective responsibility of society at large to make the environmental modifications necessary for the full participation of people with disabilities in all areas of social life. The Social Model of Disability asserts that individuals possess impairments, not disabilities (20).

Approaching MI using the Social Model of Disability reframes MI from an attribute of an individual to a socially created, complex collection of conditions, created by the social environment. Approached in this way, MI is a public health challenge, and the phenomenon of MI requires not only individual care but also social action: society needs to share responsibility for the moral challenges and social conditions MI reveals. This approach supports veteran reintegration in a way that respects moral agency and facilitates moral health.

A shift is underway, and presently, MI is widely understood as a bio-psycho-social-spiritual “problem,” and some consensus has emerged about the value of inter-disciplinary collaboration that includes chaplains and spiritual leaders alongside therapists and others (21). As Hodgson and Carey note, some conceptualizations of MI tend to focus blame on the individual, effectively exonerating institutional and public liability for harmful consequences, and chaplain involvement has brought a needed shift in attention towards community engagement, public health, and shared moral responsibility (22). Early conceptualizations of MI have been clarified, with exposure to potentially morally injurious events (PMIEs), typically involving values transgressions by self or others, now distinguished from outcomes and impacts on individual functioning, as in the Moral Injury Outcome Scale (23). To help assess the severity of impact, Gosling et al. offers a Spectrum of Moral Harm (24), Grimell and Nilsson the Integrated Moral Distress and Injury Scale (25), and VaanderWeele et al. the Moral Trauma Spectrum (26). The DSM-5, while careful not to misrepresent moral problems as illnesses or disorders, now includes MI under the “Moral, Religious, or Spiritual Problem” Z code, with moral problems defined as “experiences that disrupt one’s understanding of right and wrong, or sense of goodness of oneself, others or institutions” (27). Mattson et al. believe the new code “legitimizes an otherwise stigmatized and under-recognized aspect of human suffering, equipping both clinician and patient with necessary language” (28). Mattson et al. discuss The Moral Problem Framework (MPF) which they believe “defines moral dilemmas, distress”, and injury in a sufficiently expansive way, to include the broad range of complex moral situations that could lead to morally laden distress. Mattson et al. see the new DSM Z code as progress, fitting MI along with moral dilemmas, and moral distress within the MPF, and assert MI as “inherently distressing and possibly impairing to individual functioning.” Koenig et al. argue MI “is best conceptualized as a dimensional construct within a broader spectrum of moral trauma” (29).

Even so, MI continues to be an emerging construct and maintaining an experimental mind-set when approaching MI seems imperative. Houle et al. find key debates endure, especially concerning defining PMIEs, the role of betrayal, subjectivity, contextual factors, and blame, as well as severity and thresholds for functional impairment (30). On the one hand, the MPF supports the conceptualization of moral health offered here. The MPF allows space for “legitimate moral pain,” affirming, rightly, that all moral pain does not lead to injury. In the MPF, the experience of moral pain of one kind or another, be it, “moral frustration, moral distress, or moral trauma” is a necessary step towards the “inherently morally distressing and possibly impairing to individual functioning” condition that Mattson et al. assert is MI. On the other hand, the MPF, by placing all MI under the umbrella of moral problems, fails to capture the full range of moral challenges that might give rise to unpleasant moral emotions, that might lead to something like “moral distress,” and might contribute to impairments in moral functioning. In this way, the MPF is not expansive enough. Rather than equipping clinicians with necessary language, for reasons discussed here, the “MI as moral problem” approach subsequently diminishes the potential utility of the modified Z Code.

## Moral disengagement, moral exploitation, and moral overload

The degree to which the people whose society authorizes the kind of collective harm that is military force are morally responsible for that harm is a matter of debate, as is the degree to which military servicemembers who perpetrate harm on behalf of their society are morally responsible for their participation when state violence might be unjust or even criminal. Crawford discusses kinds and levels of moral responsibility for military atrocity, noting for example, citizens in more democratic states like the US, bear greater moral responsibility for state violence than residents of totalitarian regimes, even asserting the US public has causal and moral responsibility for not only the authorization of war or military force but also for the ways in which the war is conducted or that force is executed (31). Crawford asserts “Until we address the collective features that produce systemic atrocities, it is unlikely that they will end” (32). The language of “atrocity” needs definition. For Card, atrocity is foreseeable, intolerable harm caused by culpable wrongdoing (33). While all military force may not meet the threshold of atrocity as defined by Card, thinking about PMIEs through Card’s atrocity paradigm reveals the collective and agentic nature of the harm generative of what is often called MI. The mechanisms at work preventing people from recognizing and addressing these collective features that produce systemic atrocities are serious moral challenges. Here we consider how the mechanisms of moral disengagement and moral exploitation operate to conceal and displace moral responsibility, and how they give rise to an impairment in individual functioning we might call moral overload.

Bandura describes moral disengagement as the process whereby moral agents selectively apply moral self-sanctions—euphemistic language, advantageous comparison, displacement of responsibility, diffusion of responsibility, disregard, distortion, and denial of harmful effects—to avoid owning and acknowledging personal contribution to harm and feel good about themselves, which allows the harm to perpetuate (34). Robillard and Strawser offer the separate but related concept of moral exploitation, when moral agents “unfairly burden someone with added moral responsibility or moral decision-making” arguing “the moral exploitation of soldiers provides one plausible explanatory backdrop as to how moral injury occurs and eventuates” (35). Robillard and Strawser also assert that in the US context of an all-volunteer force, military servicemembers are morally responsible not only for their personal conduct in warfighting, but also for the act of participating in a war that is determined to be unjust or criminal, which adds to their moral dilemmas and *moral residue*, or “dirty hands”.

In the US context, the moral challenges of moral disengagement and moral exploitation prevent people from serious moral reckoning with harmful consequences of the US use of military force. People who morally disengage and morally exploit others impede moral health and moral functioning. They undermine moral security. They create conditions where morally engaged people who should and do feel unpleasant moral emotions might find those emotions persist and escalate into impairing and incapacitating injury. So, these impediments to moral health and moral functioning can and should be addressed. When they are, and moral community emerges, then the moral agent at risk of developing MI, understood as “inherently distressing and possibly impairing to individual functioning” will find in that moral community greater capacity to stay engaged and bear unpleasant moral emotions well without succumbing to impairment or injury. When moral disengagement and moral exploitation persist, so should the unpleasant moral emotions of the morally engaged person, like the canary in the coalmine, warning of the moral challenges the community needs to face to adapt and change.

A moral agent with unimpaired moral functioning who faces a PMIE and morally engages will initially feel the challenge of a moral dilemma and experience unpleasant moral emotions, which may even become moral residue, a sense of “dirty hands,” or a “moral distress,” which might lead to some kind of impairment in individual functioning, that might meet some threshold of MI, with community and public health implications. If that moral agent faces the PMIE alone, without the social conditions generative of moral security, such as a moral community willing to own and acknowledge its own violence, then that moral agent may become overwhelmed by guilt and shame, stuck in despair or resentment, bearing the burden of responsibility displaced by the collective, experiencing those emotions with such persistence and severity, that agent might suffer an impairment to individual functioning. A moral agent who assumes more than their fair share of responsibility for harmful conduct assumes moral burdens that belong to others and might experience unpleasant moral emotions to such a degree it crosses some threshold into impairment. When that impairing to individual functioning includes an impairing to moral functioning, we apply the concept moral overload (36) to name this kind of impairment. Conceptualizing this type of impairment as MI seems to be within the scope of current literature on MI. How to determine whether an impairment to individual functioning constitutes an impairment in moral functioning is an important process, helped by the above conceptualization of moral health and moral functioning. The state of being in moral overload is both inherently distressing and impairing to individual moral (and quite possibly) psychological functioning. The state of moral overload does not occur in a vacuum. When people in US society avoid unpleasant moral emotions by employing mechanisms of moral disengagement or engaging in moral exploitation, they likely prolong and exacerbate veteran’s unpleasant moral emotions, contributing to the harmful condition of moral overload.

## Moral engagement group

The MEG (previously known as the MIG) is a 12-week group co-facilitated by a chaplain and a psychotherapist with a community healing ceremony on the 10th week (See **Table 1**). The MEG engages veterans and their community around various moral challenges related to military service. The MEG community healing ceremony is a specific communal practice generative of moral security. During the ceremony, veterans are invited to give *testimony* (37) to a community willing to (at least) share responsibility and (possibly) own and acknowledge complicity in violence. As May expressed: “People should see themselves as sharing responsibility for various harms perpetrated by, or occurring within, their communities” (38) and, in the context of the ceremony, people do.

**Table 1:** Overview of the 12 week moral engagement group.

Week	Topic
1.	Introductions & Overview
2.	Dimension of Pain: Moral/Spiritual/Religious/Soul
3.	Moral Values & Moral Dilemmas
4.	Moral Emotions
5.	Moral Disengagement & Engagement
6.	Religious & Spiritual Struggle
7.	Social Contract, Moral Exploitation
8.	Sharing Responsibility by the Community Healing Ceremony
9.	Practice Testimony
10.	Community Ceremony
11.	Celebration, Reflection
12.	Closure, Next steps

Over the past decade, facilitation teams have offered 18 cohorts, 100 veterans have completed the program, and over one thousand people participated in ceremonies. In this way, the MEG has been doing the work of *moral repair* (39), understood as restoring moral health and moral functioning as described above, and restoring or creating hope and trust in a shared sense of value and responsibility.

From the start, the MEG has aimed for moral coherence by framing MI as an adaptive challenge (40). To further clarify the distinction, Heifetz et al. offer the following definitions of adaptive challenges and technical problems:

adaptive challenge: The gap between the values people stand for (that constitute thriving) and the reality that they face (their current lack of capacity to realize those values in their environment)

technical problem: Problems that can be diagnosed and solved, generally within a short time frame, by applying established know-how and procedures. Technical problems are amenable to authoritative expertise and management of routine processes (41).

The MEG does not frame MI as a technical moral *problem*, but as an adaptive moral *challenge*, making the following distinction: moral challenges occur when something morally hard is happening that invites growth; moral problems occur when something morally wrong is happening that needs to be fixed. For sure, moral problems thus defined do occur in the context of military service and are often the PMIEs that give rise to unpleasant moral emotions. People can experience a vast range of moral challenges as well as a range of unpleasant yet healthy moral emotions and still exhibit unimpaired moral functioning. Indeed, only a morally healthy person can recognize a problem as a *moral* problem to begin with and react to it with moral emotions, as doing so requires one possesses basic capacities and traits, like moral literacy, seriousness and sensitivity, and a willingness to apply a moral framework to a situation.

But are those unpleasant moral emotions, moral *problems*, or something else? What are the unexamined assumptions behind this use of language? If one starts from the standpoint of a Medical Model of Disability, and assumes a veteran exposed to a PMIE is presenting a moral problem, might that suggest the veteran needs to be fixed with the application of some technique, some treatment? If one starts from a public health standpoint, adopting the Social Model of Disability, and approaching MI as an adaptive challenge, then one broadens discourse in a way that normalizes moral emotions and invites sharing responsibility for disordered moral environments through collective work.

To say MI is an adaptive challenge is to say MI is not a technical problem that can be diagnosed and solved in a clinical encounter with therapeutic technique, removed from the people who contributed to the conditions in which the PMIE occurred. Although the MEG meets in a medical center and the chaplain and psychotherapist who facilitate are clinical staff, they emphasize unpleasant moral emotions from violence is normative and encourage veterans experiencing those emotions to identify not as patients but as prophets (42). The MEG invites veterans into a process of building moral community which culminates in the ceremony, where veterans claim their prophetic voice, testifying about the PMIE and the moral emotions they bear because of it, with the citizens who share responsibility for conditions which gave rise to those moral emotions.

Over the course of 12 weeks, the MEG guides veterans in the intrapersonal work of moral engagement, exploring moral values and moral emotions, building their moral literacy, empowering them with moral courage, as veterans face PMIEs together, in a safe space where moral security is assured. The MEG guides veterans in the interpersonal work of moral engagement through the community healing ceremony, which gathers family of the MEG veterans together with their therapists and doctors, as well as US citizens from the public, who are willing to share responsibility for various harm perpetrated by, or occurring within, the US military. To be clear, this is not an approach where unimpaired civilians meet impaired veterans to help fix their MI. Rather, the approach assumes everyone in the room, including the veteran’s chaplain and psychotherapist, is dealing with moral challenges and the only way to arrive at moral health is mutual aid and reciprocity through a process of sharing responsibility. Veterans lead this process, assuming the role of prophet, by offering testimony about various harms and their moral impact, and chaplains lead the community in a guided response which includes spoken words, symbolic acts, and ritual. This process builds moral community where moral emotions are fully experienced, moral values are embodied, and moral courage is exemplified. This process builds individual and collective capacities for facing moral challenges together. What emerges is a community living with greater moral health. Veterans express relief from moral burdens, increased intimacy among members of their family, and increased safety and trust with VA providers. US citizens express greater awareness of various harms perpetrated by, or occurring within, their communities, and publicly commit to share responsibility for those harms, in the lives of veterans and in places where veterans have unleashed violence and address the deeper wells of violence in US systems and culture.

## Discussion

From the definitions of moral health and moral functioning offered above, we can examine current conceptualizations of MI and assess their coherence. After conceptualizing moral health, Talbert and Wolfendale offer: “moral injury can occur when one’s normative competence is undermined, and/or when social conditions undermine one’s moral security and one’s standing to be treated as an equal member of the moral community” (43). Talbert and Wolfendale challenge the coherence of existing definitions of MI, by expanding the range of possible moral challenges that can give rise to impairments in moral functioning.

Many questions arise. When Mattson et al. define MI as “possibly impairing to individual functioning” are they referring to an impairing to moral functioning, or perhaps just an impairing to physical or psychological functioning? Based upon the above definition of moral functioning, when a moral agent does not experience unpleasant moral emotions, even though those emotions are an appropriate response, or does not give due moral regard to others, or does not accept moral accountability, even though the person may be complicit in harm, then there exists an impairment in moral functioning. If the absence of appropriate moral emotion is evidence of impaired moral functioning, then is that impaired moral functioning moral injury? Naming it as such challenges existing definitions, that regard MI as “inherently distressing.” Furthermore, MI defined as “possibly impairing” leaves the threshold between appropriate moral emotion, “moral distress,” and MI unclear. If impairment is the key threshold between healthy moral functioning and injury, then what exactly is impaired? Where is the impairment located, in the individual (medical model) or the community (social model)? MI defined as “possibly impairing to individual functioning” is not the same as “possibly impairing to moral functioning.” Without a conceptualization of moral health and moral functioning, current conceptualizations of MI could be morally incoherent and possibly harmful to moral health.

Miller observed “the moral dimension to psychotherapy is central to the therapeutic task itself” and urged clinicians to address moral concerns by approaching psychology and psychotherapy as moral engagement (44). Kinghorn offers psychiatry is a “discipline of moral engagement and discernment” and psychiatrists must “cultivate practices for inhabiting that space [of moral engagement] in a morally transparent, self-questioning, and responsible way” (45). Therapists could approach psychotherapy as moral engagement by acknowledging to a veteran client all the way people, including the therapist herself, are complicit in, and therefore morally accountable for, actions they contribute to even though others (like the veteran client) may be the principal agents. Therapists could approach psychotherapy as moral engagement by conceptualizing moral health as a relational and community achievement and collaborate with chaplains and spiritual leaders to create encounters where moral security is assured, moral burdens are shared, and a veteran’s family and community is included in the process of moral repair.

On the one hand, clinicians who are morally engaged approach MI with The Social Model of Disability, and can conceptualize moral health and moral functioning, will provide veterans with access to the necessary moral security and moral community to progress towards moral health. On the other hand, clinicians who are morally disengaged, stuck in the Medical Model of Disability, and lack a coherent conceptualization of moral functioning, may impede access and do moral harm.

Consider the following four scenarios. First, a veteran is suffering because they are a morally functioning moral agent without access to the moral security of moral community and is told by a provider “you have moral injury,” and the veteran understands that to mean, “I have an impairment in my moral functioning,” when that is not the case. Second, a veteran, bearing appropriate guilt and seeking moral security and moral regard, approaches a provider hoping to disclose a moral challenge from a military atrocity in which the provider herself is implicated, albeit indirectly, and at first encounter is told by that provider, “thank you for your service.” Third, a veteran, upon disclosing appropriate guilt to a provider, for an atrocity for which he is blameworthy is told, “you are not responsible for that, you did what you had to do.” In the first case, the provider risks misleading the veteran as to where the actual problem lies. In the latter two cases, the provider is employing mechanisms of moral disengagement and failing to provide appropriate moral regard, to the detriment of the veteran’s moral health.

Consider a fourth scenario: a veteran describes a sexual encounter in which he perpetrated sexual assault, but refuses to acknowledge any wrongdoing, instead employing mechanisms of moral disengagement like displacing responsibility to the victim and minimizing the harmful impact. The veteran reports no unpleasant moral emotion or psychological distress and thus does not meet the “inherently distressing” MI criteria put forth by Mattson et al., even though that veteran has impaired moral functioning, by definitions offered here. If the provider applies the DSM-5 definition of moral problems (“experiences that disrupt one’s understanding of right and wrong, or sense of goodness of oneself, others or institutions”) and accepts the veteran’s subjective understanding of right and wrong, then there is no moral problem here. Yet when a person acts in a way that is objectively wrong and does not experience unpleasant moral emotion, either because they lack certain capacities and traits, or because they are willfully employing mechanisms of moral disengagement, then that constitutes impaired moral functioning. Such an encounter presents a moral challenge to the clinician—the challenge to collude with the veteran in moral disengagement, or to be morally engaged and address the veteran’s moral disengagement as an impairment in moral functioning. In this case, impairment is the inability or unwillingness to recognize a wrongdoing and respond with appropriate emotion and moral behavior, whether due to moral illiteracy, moral insensitivity, moral unseriousness, moral complacency, moral cowardice, or some combination of these.

Veterans experiencing unpleasant moral emotions as a consequence of their military service need access to a morally engaged community, including morally engaged psychotherapists and psychiatrists, willing to face moral challenges, provide moral security, share responsibility for moral burdens, including, where appropriate, complicity and moral accountability. Likewise, people who ought to experience unpleasant moral emotions, because they have blameworthy moral responsibility, but do not, also need access to a morally engaged community willing to hold them morally accountable, and provide a hopeful vision and role model of moral health and moral functioning. Without such moral community, veterans experiencing unpleasant moral emotions are at greater risk of moral overload, or other impairments in individual functioning, and the veteran’s community misses the opportunity to face moral challenges and thrive.

## Limitations and recommendations

MEG is one among several MI protocols currently engaged by co-facilitation teams of chaplains and psychotherapists in VA to address potentially morally injurious experiences (46) however MEG is unique because of the focus on morally engaged and contextually informed care through the community healing ceremony component. This paper has offered theoretical foundations of a community intervention (MEG) for specific population (US veterans) in a specific context (US society) and recommends a conceptualization of moral health and moral functioning based in moral community. This paper also recommends more comprehensive MI care by approaching MI as an adaptive moral challenge and public health issue through the Social Model of Disability, which creates space to name and address the social conditions of moral disengagement and moral exploitation. So far, the MEG is not using any quantitative measure to assess moral health, moral engagement/disengagement or moral exploitation either in the group of veterans or in community that gathers for the ceremony. Existing scales measuring moral disengagement/engagement like the Mechanisms of Moral Disengagement Scale (MMDS) and new measures for moral health and moral exploitation could help identify impaired moral functioning and better assess the impact of interventions.
